# Atrial Mechanics in Heart Failure With Preserved Ejection Fraction: Effect of a No-Implant Interatrial Shunt

**DOI:** 10.1161/CIRCHEARTFAILURE.124.012573

**Published:** 2025-09-03

**Authors:** Michal Laufer-Perl, Nir Flint, Yaron Arbel, Fawaz Alenezi, Veraprapas Kittipibul, Dmitry Yaranov, Tamaz Shaburishvili, Rohit Amin, Marat Fudim

**Affiliations:** 1 Tel Aviv Sourasky Medical Center, Affiliated with the Gray School of Medicine, Tel Aviv University, Israel (M.L.-P., N.F., Y.A.).; 2 Duke University, Durham, NC (F.A., V.K., M.F.).; 3 Duke Clinical Research Institute, Durham, NC (V.K., M.F.).; 4 Baptist Memorial Healthcare, Memphis, TN (D.Y.).; 5 Tbilisi Heart and Vascular Clinic, Georgia (T.S.).; 6 Ascension Sacred Heart Hospital, Pensacola, FL (R.A.).

**Keywords:** atrial pressure, heart failure, humans

## Abstract

**BACKGROUND:**

The atria play an important role in the pathophysiology of heart failure with preserved ejection fraction. Decreased left atrial strain is associated with worse clinical outcomes. The impact of no-implant interatrial shunting on atrial structure and function has not been described.

**METHODS:**

We characterized the left atrial (LA) and right atrial strain-pressure relationship at rest and during exercise, before and after creation of a no-implant interatrial shunt. We included patients with New York Heart Association class II, III, or ambulatory IV heart failure with a left ventricular ejection fraction ≥40% and elevated LA wedge pressure during supine ergometry (≥25 mm Hg). Exercise hemodynamics and echocardiographic measurements were analyzed at baseline, 1 month and 6 months (echo only) following transcatheter, transeptal creation of a 7 mm no-implant interatrial shunt.

**RESULTS:**

A total of 33 patients were enrolled/included in the study. At 1 month, LA pressure at rest was significantly reduced from 19.7±7.0 to 17.2±5.0 mm Hg (*P*=0.044), and from 39.7±10.5 to 33.6±11.1 mm Hg (*P*=0.002) during exercise. Reductions in LA pressure were associated with a mean decrease of 55.4 mm Hg/W·kg in LA work (*P*<0.001). Echo measurements demonstrated significant improvements in LA reservoir strain of +4.0% (*P*=0.015) and +4.1% (*P*=0.046) at 1 and 6 months, respectively. Modest improvements were observed in LA conduit and contractile strain, with a similar overall trend in right atrial strain measurements. These findings were associated with a significant reduction in LA volumes and an increase in right atrial volume. There was no change in right atrial pressure or measures of right ventricular function.

**CONCLUSIONS:**

Hemodynamic and strain assessment in patients with heart failure with preserved ejection fraction suggests that a no-implant interatrial shunt can significantly improve the pressure-function relationship of the LA.

**REGISTRATION:**

URL: https://www.clinicaltrials.gov; Unique identifiers: NCT04583527, NCT04838353, and NCT05501652.

**WHAT IS NEW?**

- To our knowledge, this is the first study to investigate the effect of interatrial shunting on atrial strain, demonstrating that reductions in the left atrial pressure-volume relationship following the creation of a no-implant interatrial shunt significantly improve left atrial function.
- Although these findings were associated with expected increases in right atrial volume, no adverse effects on right atrial strain or right ventricular structure-function were observed, indicating safe and effective decompression of the left atrium.

**WHAT ARE THE CLINICAL IMPLICATIONS?**

- Although growing evidence supports that atrial strain is a highly sensitive index of patient symptoms and outcomes, the extent to which device interventions may impact atrial strain has only recently gained attention, in part due to emerging therapies such as interatrial shunting.
- Closer examination of the change in atrial strain in response to atrial interventions, including nonshunt device therapies, may provide significant insight into the pathophysiology driving clinical symptoms as well as identify opportunities for improving care through patient selection and device optimization.

**See Editorial by Patel and Shah**

Left atrial (LA) dysfunction plays a central role in heart failure with preserved ejection fraction (HFpEF). Derangements in LA mechanics, preceding or independent of atrial dilation, result from interstitial fibrosis or remodeling due to increased left ventricular (LV) filling pressures, leading to impaired atrial compliance and atrial dyssynchrony.^1^ Alterations in this structure-function, or more appropriately, pressure-volume relationship are further exacerbated by LA dilation, which is an important echocardiographic criterion for elevated filling pressures and has recently shown a 16% increase in the risk of atrial fibrillation for each 1 cm increase in LA diameter.^2–4^ Changes in atrial function, as assessed by atrial strain, are consistent with pressure-volume observations, and reductions in LA reservoir strain have been shown to be a strong independent predictor of outcomes in patients with HFpEF.^5,6^

Diminished atrial function promotes backward transmission of elevated LV filling pressures into the pulmonary circulation, manifesting as dyspnea and pulmonary congestion. Transcatheter interventions involving instrumentation of the atrial wall, such as ablation of atrial fibrillation, LA appendage closure, and patent foramen ovale closure, may lead to impaired atrial strain and an increase in LA stiffness.^7–11^ Specifically, altering the atrial substrate or immobilizing particular regions of the atrium may affect the atrial pressure-volume relationship. Although significant increases in LA stiffness (derivative of pressure to volume [dP/dV]) have been shown following LA appendage closure, investigations on the extent to which atrial device therapies may affect atrial function are limited.^7^

The association between percutaneous device therapies and alterations in atrial mechanics remains poorly understood, specifically in the emerging atrial interventions for HFpEF, such as interatrial shunts. Hence, the goal of the current study was to test the hypothesis that, in patients with HFpEF, a no-implant interatrial shunt can achieve hemodynamic LA decompression without adversely affecting LA mechanics or right-sided function (Figure 1).

**Figure 1.**
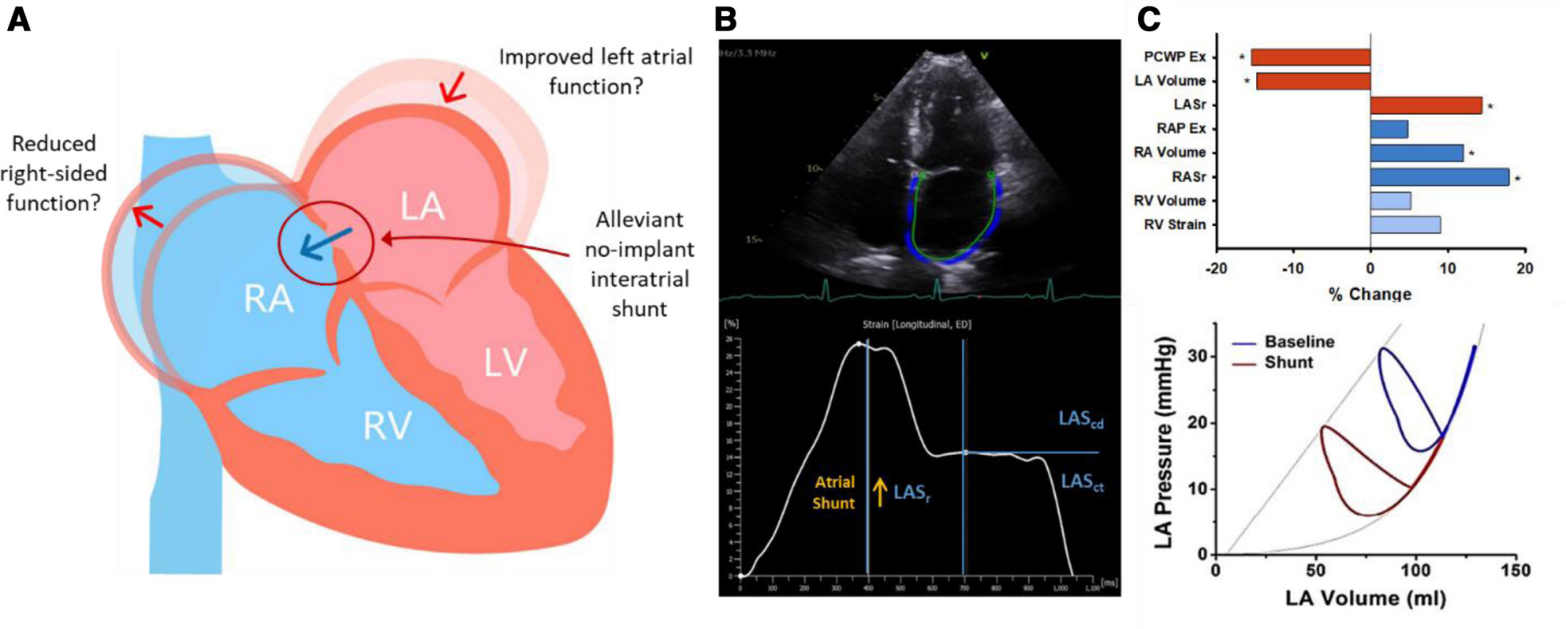
**Physiological response to a no-implant interatrial shunt. A**, Hypothesis that decompression of left atrial (LA) may improve atrial function without compromising right atrial (RA) or right ventricular (RV) performance. **B**, Representative measurement of LA strain with segmentation of strain phases. **C**, LA, RA, and RV measurements following creation of a no-implant interatrial shunt which indicate an improvement in left atrial mechanics and predicted downward left shift in the pressure-volume (PV) relationship without adversely impacting right-sided function (PV loop adapted with permission from Kaye et al^12^).

## Methods

### Study Design

The data supporting the findings of this study are available upon reasonable request and in accordance with Alleviant Medical’s data-sharing policy. Data were obtained from a pooled study cohort, ALLEVIATE-HF-1 (NCT04583527), ALLEVIATE-HF-2 (NCT04838353), and ALLEVIATE-HF-HD (NCT05501652), which were prospective, open-label, uncontrolled, and nonrandomized studies evaluating the safety and feasibility of the Alleviant system for the creation of a no-implant atrial shunt in patients with New York Heart Association classification II through ambulatory class IV and a LV ejection fraction ≥40%.

### Patient Enrollment and Procedures

After informed consent, patients underwent a clinical screening assessment, history, physical exam, and medication review, as well as Kansas City Cardiomyopathy Questionnaire assessments, 6-minute walk test, blood chemistry/hematology panels, and baseline transthoracic echocardiography. Key inclusion criteria included prior hospitalization for heart failure or exacerbation of symptoms requiring intravenous diuretics, echocardiographic evidence of diastolic dysfunction, and pulmonary capillary wedge pressure (PCWP) during supine ergometer exercise ≥25 mm Hg with a PCWP to right atrial (RA) pressure gradient ≥5 mm Hg (NCT04583527; NCT04838353; NCT05501652).^13^ Primary exclusion criteria were the presence of advanced heart failure, moderate or worse valve disease, pulmonary disease, and evidence of moderate or worse right heart dysfunction. Following screening, all subjects underwent exercise right heart catheterization, with hemodynamics measured during end-expiration and cardiac output obtained with the thermodilution method. If hemodynamic criteria were met, patients proceeded to undergo the interatrial shunt procedure, whereby a 7 mm interatrial shunt was created in the fossa ovalis using the Alleviant System (Alleviant Medical).^13^ Follow-up, including qualitative (Kansas City Cardiomyopathy Questionnaire) and functional (transthoracic echocardiography, 6-minute walk test) assessments, were performed at 1, 3, and 6-month follow-up visits, in addition to exercise hemodynamic evaluation at 1 month. Study protocols were approved by local Ethics Committees.

### Volume and Strain Measurements

For atrial and ventricular volume measurements, echo images were uploaded to the vendor-independent TomTec image arena module (REF-Version 4.6 software, Munich, Germany). LV volumes were obtained from biplane views. Right ventricular (RV) end-diastolic volume was performed using the single-plane Simpson method from the apical RV-focused view. For strain measurements, a semi-automated speckle-tracking analysis was performed using a commercial tracking software (TOMTEC arena AutoSTRAIN; TomTec Imaging Systems, Unterschleissheim, Germany). LA strain was assessed from the apical 4-chamber view, using end-diastole as the zero reference, with all strain values reported as positive. RV strain was obtained from the apical focused RV view and calculated as both free wall and total strain (4-chamber). The phases of atrial function were measured: (1) Reservoir (peak longitudinal strain); (2) Contractile (peak atrial contraction strain); (3) Conduit (difference between peak longitudinal strain and peak atrial contraction strain). LV global longitudinal strain was calculated as an average of 18 segments from the apical 2-, 3-, and 4-chamber views. Measurements were automatically tracked throughout the cardiac cycle and reviewed by the investigator. Manual corrections were performed when needed to optimize endocardial boundary tracking. All measurements were performed by a single expert strain investigator blinded to clinical status.

### Data and Statistical Analyses

The within-group response to no-implant interatrial shunting was evaluated. The analysis population was from the per-protocol population (patients who were enrolled and treated with the investigational device and evaluated at 1- and 6-month follow-up). Baseline demographics and characteristics were reported as either mean±SD or in frequencies, dependent on the variable. Sex-based or race/ethnicity-based differences were not analyzed due to sample size. Normally distributed continuous variables, including hemodynamic, volume, and strain measurements, were calculated from the change in baseline to follow-up and analyzed using a paired *t* test or repeated measures ANOVA as appropriate. Nonnormally distributed variables between groups were compared using a Wilcoxon signed-rank test. Derived 95% CIs are provided where appropriate and unless otherwise stated. In instances where follow-up data were unavailable or echo image quality was insufficient, the subject was excluded from the analysis, as indicated in the respective tables.

## Results

### Population Sample and Baseline Characteristics

Baseline characteristics are provided in Table 1. Between August 2020 and July 2022, 33 patients were enrolled in the studies. Technical success was achieved in all patients, with no procedural adverse events. The mean age of participants was 68±9 years; 67% were women (n=22), and the majority (76%) were at New York Heart Association class III (n=25) with a prior hospitalization for heart failure (88%). All patients were consistent with definitions of HFpEF based on LV ejection fraction, elevations in resting and exercise PCWP, and presented with alterations in indices of functional capacity (6-minute walk test), quality of life (Kansas City Cardiomyopathy Questionnaire), and congestion (NT-proBNP [N-terminal pro-B-type natriuretic peptide]). One patient was lost to follow-up.

**Table 1.**
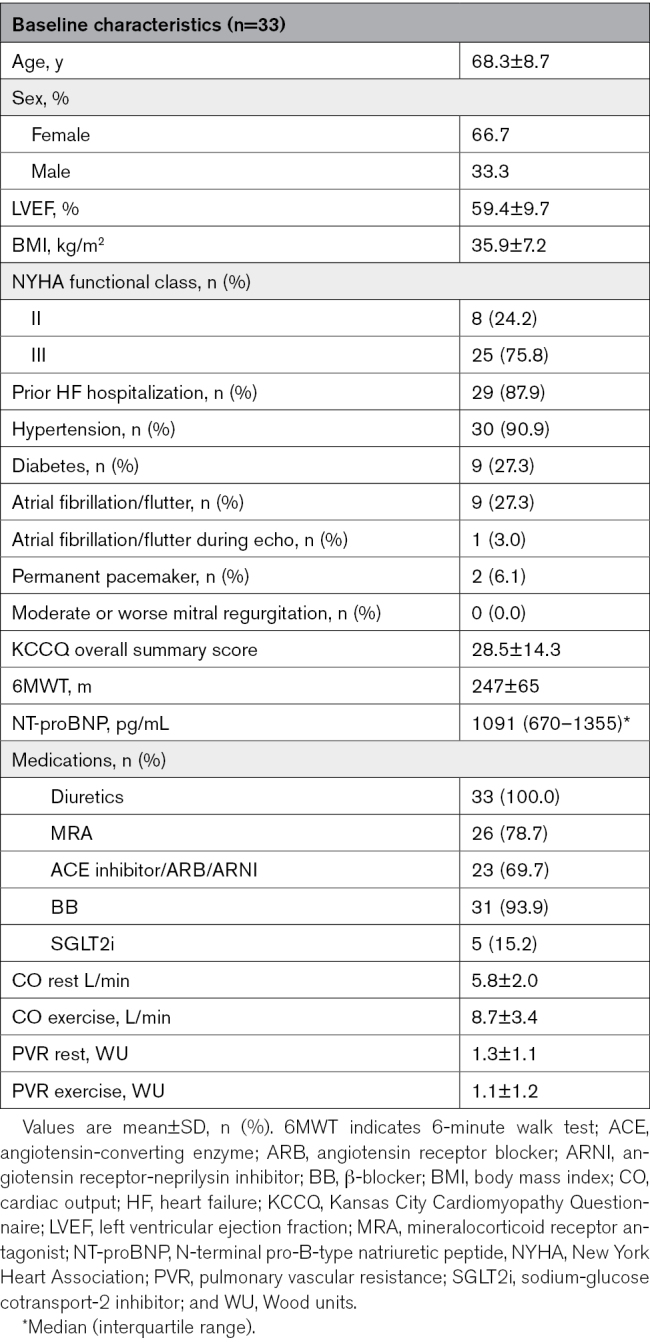
Baseline Characteristics

### Effect of Shunt on Atrial Pressure-Volume

**Figure 2.**
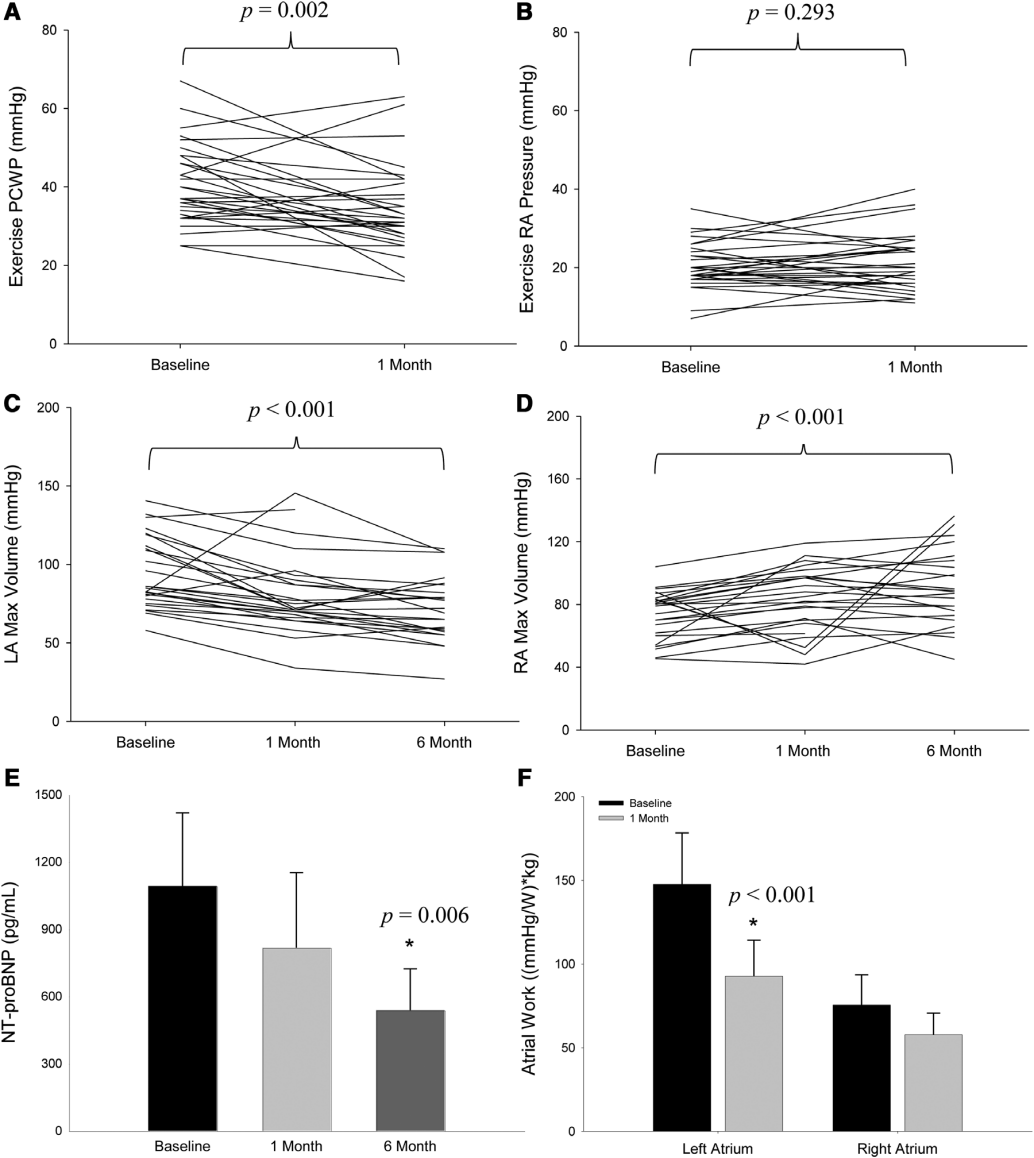
**Atrial decompression. A**, Pulmonary capillary wedge pressure (PCWP) peak exercise. **B**, Right atrial (RA) peak exercise pressure. **C**, Left atrial (LA) max volume. **D**, RA max volume. **E**, Median NT-proBNP (N-terminal pro-B-type natriuretic peptide). **F**, Mean atrial work. Significant reductions in PCWP and LA volume were observed through 6 months post-shunt creation, with increases in RA volume but not RA pressure. Consistent with decompression, NT-proBNP and atrial work were decreased through 6-month follow-up. Error bars represent 95% CIs. * signifies a *P*<0.05 vs baseline.

Hemodynamic measures from baseline to 1 month are shown in Table 2 and Figure 2. PCWP decreased significantly at rest and during peak exercise at 1 month following creation of a no-implant interatrial shunt. Concordant with a reduction in LA pressure, LA maximum and minimum volumes were significantly reduced, suggesting a downward left shift in the pressure-volume relationship which aligns with improved measures of compliance (LA reservoir strain/E/e′). The change in LA volumes were proportional to significant increases in RA maximum and minimum volumes. However, no significant postshunt elevations in RA pressure were observed at rest or exercise, consistent with effective shunting, accommodation of the increase in blood flow, and a rightward-only shift in the pressure-volume relationship.

**Table 2.**
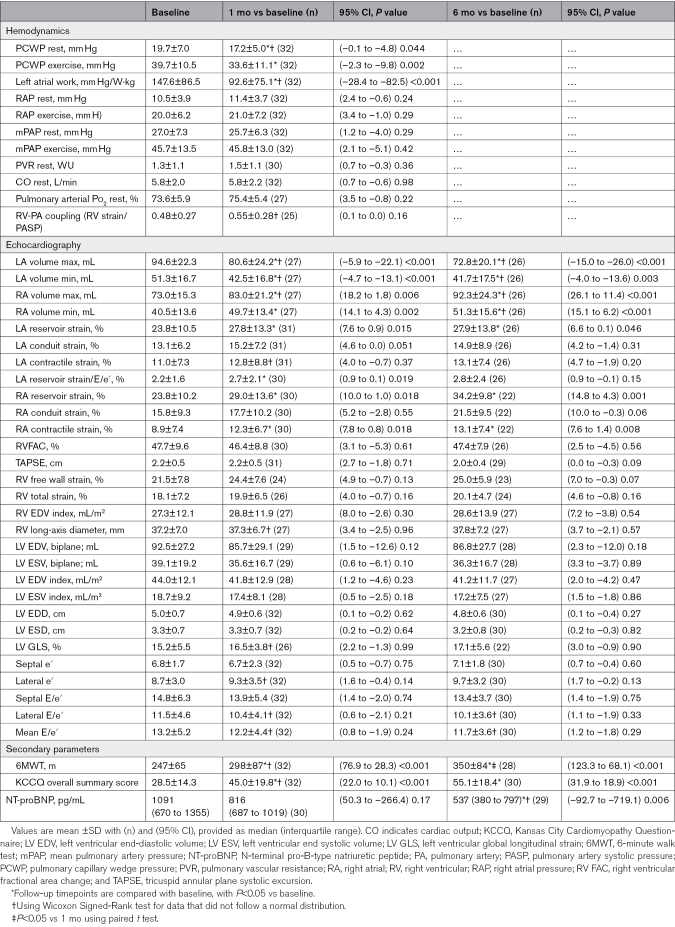
Hemodynamic, Echo, and Functional Parameters

Observed differences in the effect of shunting on the left versus right atria corresponded with measurements of atrial work (pressure per Watts of exercise and weight), whereby a significant reduction in work was observed only for the LA. The attenuation in LA work was associated with a marked reduction in NT-proBNP, decreasing from a median of 1091 (IQR, 670–1355) pg/mL at baseline to 537 (IQR, 380–797) pg/mL at 6 months. Although pressure measurements were only obtained at 1 month post-shunt, 6-month reductions in volume, work, and NT-proBNP suggest sustained decompression of the LA through a no-implant approach to interatrial shunting.

### Atrial Function in Response to the Shunt

**Figure 3.**
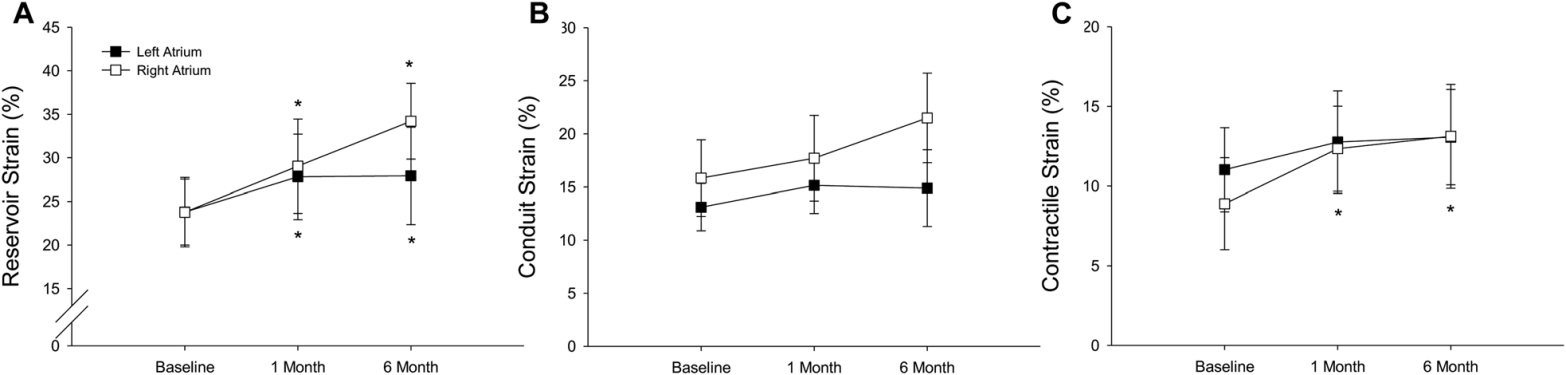
**Atrial strain. A**, Significant increases in reservoir strain were observed at 1 month (left atrial [LA] *P*=0.015, right atrial [RA] *P*=0.018) and sustained at 6 months (LA *P*=0.046, RA *P*=0.001). **B**, Effects on LA conduit strain were more modest. **C**, Only RA contractile strain increased during the 6-month follow-up period. Error bars represent 95% CIs. * signifies a *P*<0.05 vs baseline.

Atrial reservoir, contractile, and conduit strain were measured at baseline, 1- and 6-month timepoints to evaluate the effect of interatrial shunting on atrial function as shown in Figure 3. For the LA, reservoir strain was significantly increased from baseline to 1-month and 6-month follow-ups. LA conduit strain also increased but was more modest and did not reach significance at 1-month or 6-month. No differences were observed in the LA contractile strain. Similar changes were observed in RA strain parameters, including a significant increase in RA reservoir strain at both follow-up timepoints. In contrast to the LA, RA contractile strain increased and combined with increases in RA reservoir strain and volume, suggests physiological adaptation to increased RA preload. Only a modest difference was noted in LA and RA conduit strains.

**Figure 4.**
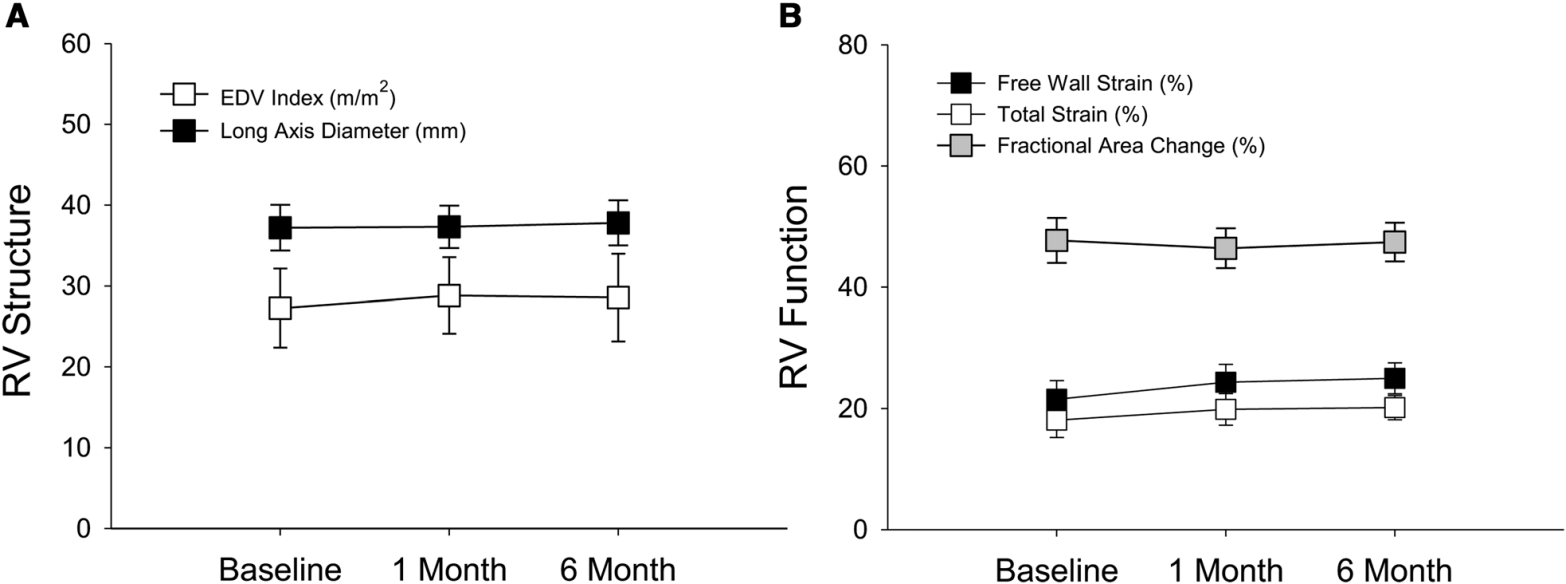
**Right-sided function.** No observable changes were noted in right ventricular (RV) structural (**A**) or functional (**B**) parameters from baseline to 6 months, including RV end-diastolic volume (EDV) index, RV long-axis diameter, RV strain (free wall, total), and RV fractional area change (FAC). Error bars represent 95% CIs.

No discernable changes were identified in RV structure or function, including RV end-diastolic volume index, RV long-axis diameter, RV free wall strain, RV total strain, and RV fractional area, as shown in Figure 4. RV-PA coupling (RV strain/PA systolic pressure) improved modestly but did not reach significance. Moreover, LV diastolic and systolic volumes, as well as global longitudinal strain, were also unaffected by shunting. Similarly, only modest improvements were observed in septal e′, lateral e′ or mean E/e′. However, significant improvements were observed in the clinical functional and qualitative assessments as shown in table 2 Specifically, patient 6-minute walk test increased by ≈30% while Kansas City Cardiomyopathy Questionnaire overall summary score nearly doubled at 6 months.

## Discussion

This investigation tested the hypothesis that a no-implant interatrial shunt could achieve hemodynamic LA decompression in patients with HFpEF, without adversely affecting LA/RA mechanics. In addition to a known reduction in PCWP both at rest and during peak exercise following the creation of a no-implant interatrial shunt,^13^ results from this analysis demonstrate a significant reduction in LA volume, LA work, and an improvement in LA mechanics through an increase in LA reservoir strain. Importantly, the absence of a decline in right-sided functional parameters, including RA strain, RV free wall strain, RV total strain, tricuspid annular plane systolic excursion, and RV fractional area change suggest a favorable risk-benefit to decompression of the left atrium.

Unlike previous studies using atrial volumetric indices as surrogates for atrial mechanics, this study is the first to use atrial strain to assess changes in atrial mechanics in response to no-implant interatrial shunting therapy. Atrial strain is an established marker of atrial mechanics, superior to volumetric indices, mainly because of its ability to specifically assess each phase of the atrial cardiac cycle. Among patients at risk for LV diastolic dysfunction, the prevalence of abnormal LA strain was approximately 2-fold that of abnormal LA volume index (63% versus 34%).^14^ Even among patients with normal LA volume index, about 30% had abnormal LA strain.

The study demonstrates favorable hemodynamic effects of no-implant interatrial shunting, by providing durable and effective decompression of the LA, leading to a sustained reduction in LA volume and a predictable increase in RA volume. Although an increase in RA volume occurred, we did not observe significant changes in RA pressure or RV functional parameters suggesting accommodation of the pulmonary vasculature to increased blood flow. In addition, the increase in RA maximum volume, minimum volume, reservoir strain and contractile strain was not associated with an increase in the atrial work of RA which we hypothesize is the result of changes in preload leading to a rightward shift in the pressure-volume relationship.

Atrial septal interventions could inadvertently lead to unfavorable effects on atrial mechanics. In patients undergoing atrial septal defect closure, despite a significant decrease in LA volume, LA strain tends to decrease over time, with this effect being more pronounced following percutaneous device closure compared with surgical closure.^10,15^ This observation is likely attributed to the bulky closure device restricting normal atrial motion, whereby pressure and wall stress become elevated at a given volume. Our hypothesis is partially supported by a study demonstrating that impaired peak regional atrial longitudinal strain was confined to the atrial septum near the device, while the lateral wall strain remained unaffected.^16^ This study also found a significant correlation between larger closure devices and a greater reduction in LA strain.^17^ Similarly, percutaneous patent foramen ovale closure devices are associated with postimplant atrial fibrillation, as well as impaired LA strain.^18,19^ Applying this notion to interatrial shunting, it is plausible that device-based shunting therapy could similarly impair atrial mechanics. Therefore, a zero-footprint technique might be preferable. Accordingly, our study demonstrates an improvement in LA and RA reservoir strain following no-implant interatrial shunting, supporting our hypothesis that the no-implant interatrial shunt did not negatively affect the RA/LA mechanics. Moreover, past studies have demonstrated that the reservoir phase holds the strongest reliability and predictive value for cardiovascular outcomes.^20,21^

The impact of interatrial shunting on atrial function and cardiac structure, beyond hemodynamic improvement, has also recently gained attention due to published results from 2 pivotal investigations with permanent implant devices in the septum: RELIEVE-HF (Reducing Lung Congestion Symptoms in Advanced Heart Failure) and REDUCE LAP-HF II (Reduce Elevated Left Atrial Pressure in Patients With Heart Failure).^22,23^ In particular, shunt treatment in the HFpEF cohort of RELIEVE-HF did not demonstrate a reduction in LA volume with minimal changes in RV fractional area, tricuspid annular plane systolic excursion, and RV end-diastolic area index. In contrast, patients in REDUCE LAP-HF II responder cohort demonstrated modest to significant reductions in LA volumes concurrent with preserved RA pressure and tricuspid annular plane systolic excursion despite increases in RA and RV volumes. Given the positive treatment effect reported in REDUCE LAP-HF II (3-year WIN ratio=1.6, *P*=0.006), it appears that reductions in LA pressure-volume even in the presence of increases in RA and RV volumes are associated with a positive treatment effect so long as right-sided function is preserved. Moreover, LA and RA reservoir strain were significantly higher at baseline in the responder versus nonresponder cohort of REDUCE LAP-HF II, suggesting that atrial function as assessed by strain analysis may be a key determinant of a positive response to shunting. Comparatively, data from the present study indicate that a no-implant approach to interatrial shunting which preserves septal motion may lead to greater improvements in LA structure (ie, pressure-volume), function (ie, strain), and hence clinical benefit. However, a more comprehensive follow-up analysis including strain from these and ongoing pivotal studies is required to better understand the determinants of shunt responders.

Standardized assessment of atrial mechanics, alongside hemodynamic evaluation, is warranted in future studies examining interatrial shunt therapy and other atrial interventions. This approach would allow for the early identification of intervention effects on atrial function and ensure comparability across studies. Since atrial dysfunction is a well-established predictor of poor outcomes in HFpEF, undetected atrial dysfunction unintentionally caused by atrial septal interventions could contribute to the lack of clinical improvement observed in some studies.^24^ Detecting preclinical atrial dysfunction using sensitive markers such as atrial strain may allow for early interventions before irreversible atrial remodeling occurs, potentially preventing or slowing the progression of the HFpEF syndrome.

## Limitations

This study has some limitations. First, the study is an open-label, single-arm design. The absence of a control group limits our ability to conclusively attribute the observed improvements solely to the intervention. However, given that LA strain is known to deteriorate in patients with HFpEF,^25^ the improvement in LA reservoir strain strengthens the hypothesis for a beneficial effect of the no-implant interatrial shunt. A prospective, multicenter, randomized, sham-controlled, double-blinded study is currently ongoing and will provide stronger insight into the effect of interatrial shunting on atrial mechanics (Alleviant ALLAY-HF: NCT05685303). Second, the small sample size reduces the power of our results and necessitates caution in their interpretation, in addition to a less comprehensive strain analysis which typically includes both 4- and 2-chamber images. Third, with the follow-up limited to only 6 months, the mid- or long-term effects of the no-implant interatrial shunting on atrial mechanics are unknown. Finally, despite a comprehensive assessment of LV structural and functional parameters, the assessment of RV focused more on functional parameters (eg, RV strain, tricuspid annular plane systolic excursion, and RV fractional area) with structural evaluation limited to RV end-diastolic volume index.

## Conclusions

To our knowledge, this is the first study to assess the effect of interatrial shunting on atrial strain, with results suggesting that the no-implant interatrial shunt created by the Alleviant System (Alleviant Medical) may improve the LA pressure-volume relationship in HFpEF patients without detrimentally affecting LA, RA, or ventricular mechanics. However, future research in a larger, randomized cohort is required, including regional strain analysis, to confirm these findings and evaluate the long-term benefits and risks on atrial function with various approaches to interatrial shunting (implant-free or with implant) under investigation.

## ARTICLE INFORMATION

### Sources of Funding

This analysis was supported by Alleviant Medical.

### Disclosures

Dr Laufer-Perl has received speaker honoraria from BI, AstraZeneca, Novartis, Pfizer, Novonordisk, Abbvie, Bayer, Medison, V-wave, and Alleviant; has served as a consultant to BI, Astrazeneca, Alleviant, and Bayer; and receives research grants from BI, AstraZeneca, Novartis, Pfizer, and Bayer. Dr Flint has received speaker honoraria from BI and Edwards LifeSciences; and has served as a consultant to Edwards LifeSciences, Truleaf Medical, and Restore Medical. Dr Fudim received consulting fees from Abbott, Ajax, Alio Health, Alleviant, Artha, Audicor, AxonTherapies, Bayer, Bodyguide, Bodyport, Boston Scientific, Broadview, Cadence, Cardioflow, Cardionomics, CVRx, Daxor, Deerfield Catalyst, Edwards LifeSciences, Echosens, EKO, Feldschuh Foundation, Fire1, FutureCardia, Galvani, Gradient, Hatteras, HemodynamiQ, Impulse Dynamics, Intershunt, Medtronic, Merck, NovoNordisk, NucleusRx, Orchestra, Pharmacosmos, PreHealth, Presidio, Procyreon, ReCor, SCPharma, Shifamed, Splendo, Summacor, SyMap, Verily, Vironix, Viscardia, and Zoll. The other authors report no conflicts.

## Supplementary Material
